# Topical Simvastatin Improves Lesions of Diffuse Normolipemic Plane Xanthoma by Inhibiting Foam Cell Pyroptosis

**DOI:** 10.3389/fimmu.2022.865704

**Published:** 2022-05-10

**Authors:** Siyuan Zha, Xia Yu, Xiaoxiao Wang, Yan Gu, Yidong Tan, Ying Lu, Zhirong Yao

**Affiliations:** ^1^ Department of Dermatology, Xinhua Hospital, School of Medicine, Shanghai Jiao Tong University, Shanghai, China; ^2^ Institute of Dermatology, Shanghai Jiao Tong University School of Medicine, Shanghai, China

**Keywords:** diffuse plane xanthoma, foam cells, oxidized-LDL, pyroptosis, simvastatin

## Abstract

Xanthoma pathogenesis is speculated to be associated with oxidized low-density lipoprotein (ox-LDL) deposition, although this remains unclear. Most patients with diffuse plane xanthomas present elevated blood lipid levels, and they benefit from treatment with oral lipid-lowering agents. However, there is no available treatment for diffuse normolipemic plane xanthoma (DNPX). In this study, for the first time, we used a topical simvastatin ointment to treat DNPX in three pediatric patients and observed favorable results. Immunofluorescence staining showed that the pyroptotic pathway was significantly attenuated after topical simvastatin application on the skin lesions of the patients. As ox-LDL deposition was observed in the lesions, we used ox-LDL to build a foam cell model *in vitro*. In the ox-LDL-induced foam cell formation, simvastatin consistently inhibited pyroptotic activation and inflammation in the macrophages. Additionally, the overexpression of nucleotide-binding oligomerization domain-like receptor protein 3 (NLRP3) or 3-hydroxy-3-methyl-glutaryl-coenzyme A (HMG-CoA) reductase (HMGCR), the known target of statins, reversed the effects of simvastatin. Moreover, gasdermin D (GSDMD) or HMGCR knockdown inhibited ox-LDL-induced pyroptosis. Furthermore, the immunoprecipitation results confirmed the interaction between NLRP3 and HMGCR, and this interaction was inhibited by simvastatin. In conclusion, we demonstrated that topical application of simvastatin ointment might be a promising treatment for DNPX skin lesions and that this therapeutic effect may be related to pyroptosis inhibition *via* HMGCR inhibition in foam cells. Moreover, xanthoma pathogenesis might be associated with ox-LDL deposition and inflammation.

## Introduction

Diffuse plane xanthoma (DPX) is clinically characterized by large, flat, plaque-like, yellow-to-orange skin lesions that are mostly symmetrically distributed over the axillae, neck, shoulders, or buttocks (1). DPX not only seriously affects the patients’ quality of life, but is also associated with systemic diseases, such as monoclonal gammopathy, which may lead to death (2–4). Histological manifestations include the infiltration of a large number of high-lipid foam cells and inflammatory cells in the dermis (5). Multinucleated giant cells (Touton giant cells), characterized by a cytoplasm with a high lipid content surrounded by rings of nuclei, have been observed (2). However, the pathogenesis of DPX remains unclear and might be associated with a local inflammation resulting from abnormal lipid deposits (6–8). It has been speculated that foam cells are formed from macrophages that excessively take up low-density lipoprotein (LDL) particles as well as the oxidative modification of LDL (ox-LDL) (6). Similarly, in atherosclerosis, histopathology has shown extensive foam cell formation and inflammatory cell infiltration. In addition, it has been recognized that atherosclerosis is caused by the formation of foam cells from macrophage phagocytosis of ox-LDL (9). Therefore, for *in vitro* studies on atherosclerosis, ox-LDL is usually used to construct foam cell models (10, 11). As there is currently no cell model for xanthoma, we referred to studies on atherosclerosis and used ox-LDL to establish a foam cell model in our study.

Most patients with DPX present elevated blood lipid levels, and treatment with oral lipid-lowering agents has promising results (12, 13). However, a small number of patients with DPX show normal blood lipid levels, known as diffuse normolipemic plane xanthoma (DNPX), for which no effective treatment is available (5, 14). Therefore, it is important to develop new therapeutic strategies.

Statins, particularly simvastatin, exhibit anti-inflammatory and immunomodulatory effects, such as inhibiting inflammation in lung diseases (15–17). In our previous study on DPX, topical simvastatin improved symptoms, resulting in a fewer and smaller lesions in patients (18).

Pyroptosis is a form of inflammatory cell death and is related to several inflammatory diseases (19–21). Through caspase-1 activation and subsequent gasdermin D (GSDMD) cleavage, the GSDMD-N-terminal (GSDMD-N) oligomerizes and forms pores in the plasma membrane (22). Thereafter, cell swelling, pyroptotic bubble formation, and membrane rupture cause massive leakage of cytosolic contents, such as the inflammatory cytokines interleukin (IL)-1β and IL-18 (23). In recent studies, ox-LDL has been shown to activate nucleotide-binding oligomerization domain-like receptor protein 3 (NLRP3), recruit apoptosis-associated speck-like protein containing a caspase recruitment domain (ASC), form inflammasomes, and induce classical pyroptosis in endothelial cells (24, 25). However, the effect of statins on ox-LDL-induced pyroptosis, especially in foam cells, is unknown.

Therefore, in this study, we examined whether topical simvastatin is useful for treating DNPX and then performed immunofluorescence staining of lesion samples to detect the pyroptotic pathway. Furthermore, by establishing an *in vitro* foam cell model, the mechanism underlying the effects of simvastatin and ox-LDL-induced pyroptosis was partially elucidated.

## Methods

### Human Samples

The study was conducted in accordance with the Declaration of Helsinki and approved by the Ethics Committee of Xinhua Hospital Affiliated to Shanghai Jiaotong University School of Medicine (XHEC-2017-104-2). Written informed consent was obtained from the patients before inclusion. Simvastatin ointment at a concentration of 1% was prepared in the laboratory of our hospital pharmacy. Three pediatric patients were treated with simvastatin ointment on the right side of the body twice per day for 6 months and the corresponding placebo ointment without simvastatin on the left side of the body as a control. Efficacy was assessed by comparing clinical photographs taken before and after treatment. Skin samples were obtained from the three patients after treatment.

### Cell Culture and Treatment

A human monocyte cell line (THP-1; ATCC, Manassas, VA, USA) was cultured in RPMI 1640 medium (Gibco, Grand Island, NY, USA). Differentiation of THP-1 monocytes into macrophages was stimulated by 24 h of incubation with 50 nM phorbol-12-myristate-13-acetate (Sigma-Aldrich, St. Louis, MO, USA), followed by 24 h of incubation in RPMI 1640 medium without phorbol-12-myristate-13-acetate. Confluent cell cultures were exposed to 20 μg/mL ox-LDL (Yeasen Biotech, Shanghai, China) for 8 h, with or without 2 μM simvastatin (Selleckchem, Houston, TX, USA).

### Cell Transfection

The cells were transfected with adenoviral vectors harboring the desired gene (Ad-RNA) for 24 h. The cells were collected 48 h after transfection or treatment with appropriate reagents. The cells were transfected with lentiviral vectors harboring short hairpin RNA (shRNA), and stable knockdown cells were selected using puromycin (Merck, Kenilworth, NJ, USA). The transcripts of Ad-NLRP3 and 3-hydroxy-3-methyl-glutaryl-coenzyme A (HMG-CoA) reductase (HMGCR) were NM_004895 and NM_000859, respectively. The sequences of the CD36 shRNAs, GSDMD shRNAs and HMGCR shRNAs were 5′-AAGGAATCCCTGTGTATAGAT-3′, 5′-GATGAGGTGCCTCCACAACTT-3′ and 5′-GACAGAATCTACACTCTCA-3′, respectively.

### Hematoxylin and Eosin and Tissue Immunofluorescence Staining

Five-micrometer-thick sections were obtained from each paraffin block and stained with hematoxylin and eosin (H&E; Abcam, Cambridge, UK). The other sections were incubated with the ox-LDL (Abcam, ab14519), NLRP3 (Abcam, ab214185), ASC (Cell Signaling Technology, Danvers, MA, USA, 13833), caspase-1 (Abcam, ab62698), GSDMD (Sigma, WH0079792M1), IL-1β (Cell Signaling Technology, 12242), and IL-18 (Abcam, ab243091) antibodies, and then incubated with fluorescent secondary antibodies (Cell Signaling Technology).

### Cytotoxicity Assay

Cytotoxicity was determined by measuring the release of lactate dehydrogenase (LDH) in the cell supernatants using an LDH cytotoxicity assay kit (Beyotime, Jiangsu, China) according to the manufacturer’s instructions.

### Hoechst 33342/Propidium Iodide Fluorescence Staining and Flow Cytometry

Pyroptosis was assessed using an apoptosis and necrosis assay kit (Beyotime) according to the manufacturer’s instructions.

### Enzyme-Linked Immunosorbent Assay

IL-1β and IL-18 levels in the cell supernatants were measured using enzyme-linked immunosorbent assay (ELISA) kits (Dakewe, Shenzhen, China) according to the manufacturer’s instructions.

### Cell Immunofluorescence Staining and Confocal Microscopy

The cells were incubated with the GSDMD-N antibody (Cell Signaling Technology, 36425) and then with the fluorescent secondary antibody (Cell Signaling Technology). Images were captured using a confocal microscope (Olympus, Tokyo, Japan).

### Western Blotting

Cellular proteins were electrophoresed and transferred onto polyvinylidene fluoride membranes (Millipore, Billerica, MA, USA), and then sequentially detected using primary antibodies, secondary antibodies, and an enhanced chemiluminescence substrate (Millipore). Antibodies against NLRP3 (15101), ASC (13833), caspase-1 (3866), cleaved caspase-1 (4199), GSDMD (93709), GSDMD-N (36425), IL-1β (12703), cleaved IL-1β (83186), and β-actin (4970) were purchased from Cell Signaling Technology. HMGCR (ab174830) antibodies were obtained from Abcam.

### Cellular Cholesterol Content

Cholesterol content in the macrophages was assessed using a cholesterol quantitation assay (Abcam) according to the manufacturer’s instructions.

### Statistical Analysis

The results are expressed as the mean ± standard error. Comparisons between groups were performed using the independent samples *t*-test. Statistical significance was set at *p* < 0.05. All experiments were performed independently, with at least three replicates.

## Results

### Topical Simvastatin Application Improves Lesions of DNPX and Inhibits Pyroptosis in the Dermis

**Figure 1:**
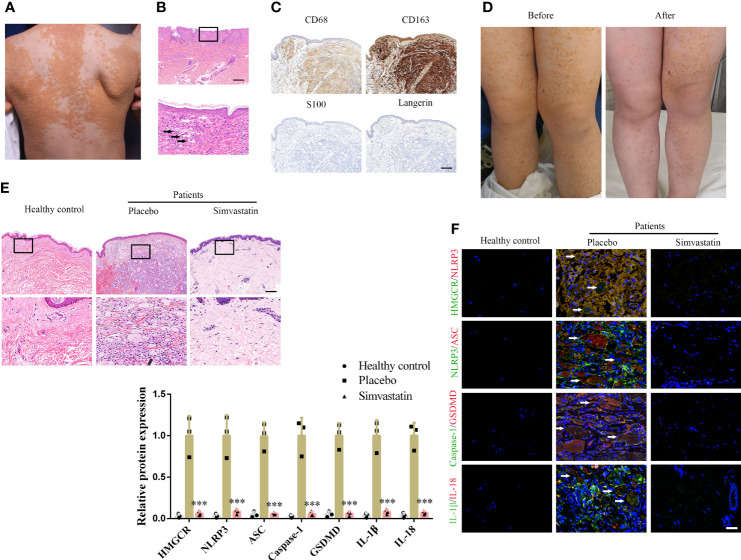
Simvastatin improves clinical manifestations and inhibits pyroptosis in the dermis. **(A)** Patient’s clinical symptoms. **(B)** Hematoxylin & eosin (H&E) staining showing foam cells (black arrow) and Touton giant cells (white arrow). Scale bar, 200 μm. **(C)** Immunohistochemical staining for diagnosis. Scale bar, 100 μm. **(D)** Patient’s clinical symptoms before and after simvastatin treatment. **(E)** H&E staining showing foam cells and inflammatory cells treated with placebo (left side of body) and simvastatin (right side of body). Scale bar, 100 μm. **(F)** Immunofluorescence staining to detect HMGCR, NLRP3, ASC, caspase-1, GSDMD, IL-1, and IL-18 levels in lesions treated with the placebo (left side of body) and simvastatin (right side of body). Arrow (foam cells). Scale bar, 25 μm. ***p < 0.001, versus the placebo.

All three patients were infants or children presenting yellowish nodules that multiplied throughout the body (**Figure 1A**). Through H&E staining, foam cells and Touton giant cells were observed in all three patients (**Figure 1B**). Immunohistochemical staining (CD68- and CD163-positive as well as S100- and Langerin-negative staining) confirmed the DNPX diagnosis (**Figure 1C**) (2). Blood lipid levels in all three patients were within the normal range and no systemic disease was found. All patients’ characteristics at baseline are presented in **Supplementary Table 1**. The severity of the lesions in the three patients significantly improved with 6-month topical simvastatin treatment (**Figure 1D**). No severe adverse events were observed except for slight erythema, which spontaneously alleviated after 3–5 days of simvastatin treatment. H&E staining also revealed fewer foam cells and inflammatory cells after simvastatin treatment. Healthy controls refer to normal peer children having skin without the related disease (**Figure 1D**). To study the mechanism underlying the effects of simvastatin, we performed immunofluorescence staining on the lesion samples. The levels of HMGCR, NLRP3, ASC, caspase-1, GSDMD, IL-1β, and IL-18 in patients with DNPX were higher than those in healthy patients and was attenuated after simvastatin treatment than those on the placebo side at the end of treatment (**Figure 1F**).

### Ox-LDL Promotes Macrophage Pyroptosis

**Figure 2:**
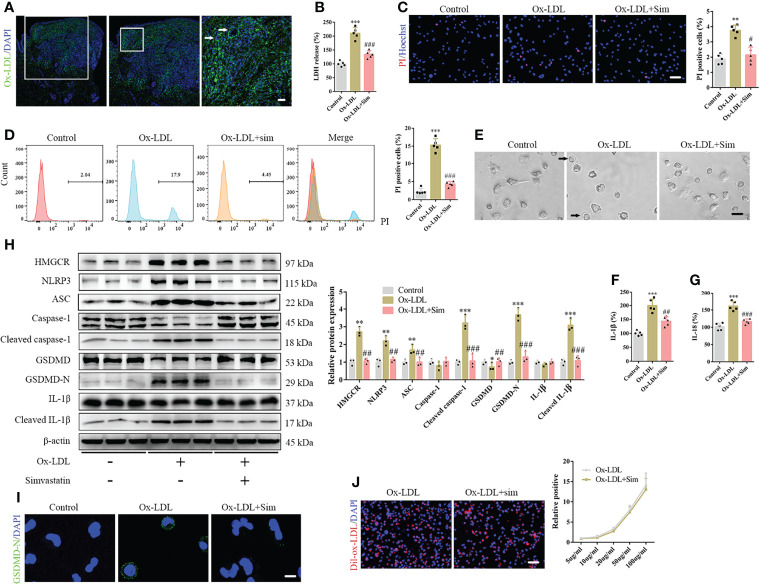
Simvastatin inhibits the classical pyroptotic pathway in foam cells. **(A)** A large amount of oxidized low-density lipoprotein (ox-LDL) is deposited in the cytoplasm of the upper dermis, and Touton giant cells were observed (arrow). **(B)** Lactate dehydrogenase release in the cell supernatants. **(C)** Hoechst 33342 (blue) and propidium iodide (red) fluorescence staining showing cell membrane integrity. Scale bar, 100 μm. **(D)** Flow cytometry for the detection of pyroptotic cells. **(E)** Pyroptotic bubbles (arrow) were observed under the microscope. Scale bar, 25 μm. **(F, G)** Results of enzyme-linked immunosorbent assay for determining IL-1β and IL-18 levels in the cell supernatants. **(H)** Western blotting showing protein abundance. **(I)** Immunofluorescence staining showing GSDMD-N expression activation. Scale bar, 12.5 μm. **(J)** Macrophage phagocytosis of Dil-ox-LDL. Scale bar, 100 μm. **p < 0.01, ***p < 0.001, versus the control. ^#^p < 0.05, ^##^p < 0.01, ^###^p < 0.001, versus the ox-LDL group.

The pathogenesis of DNPX is thought to be associated with the abnormal deposition of ox-LDL (6). Therefore, we performed immunofluorescence staining of ox-LDL. Our results showed a significant amount of ox-LDL deposition in the cytoplasm of the upper dermis, such as Touton giant cells. In contrast, a negligible amount was observed in the normal lower dermis and subcutaneous tissue (**Figure 2A**). The results are consistent with those of HE staining. Therefore, we established an *in vitro* foam cell model for macrophage phagocytosis of Dil-ox-LDL (**Supplementary Figure 1A**). Specifically, we set up a concentration gradient to stimulate macrophage response to ox-LDL. Exposure to ox-LDL enhanced LDH release into the supernatant and increased macrophage proliferation; however, ox-LDL concentrations did not fully positively correlate with the levels of released LDH and negatively correlated with macrophage proliferation, which indicates that ox-LDL not only induces macrophage pyroptosis, but also promotes macrophage proliferation (**Supplementary Figures 1B, C**). A study had reported that ox-LDL causes macrophage proliferation (26), which is consistent with our results. Therefore, we were more concerned about the release of inflammatory cytokines than macrophage proliferation in pyroptosis. Based on the results, 20 µg/mL ox-LDL was used in the subsequent experiments. We also observed pyroptotic bubbles under the microscope (**Supplementary Figure 1D**). Confocal microscopy images showed that GSDMD-N was localized to the cell membrane (**Supplementary Figure 1E**). To further confirm pyroptotic activation, we performed Hoechst/propidium iodide (PI) staining; the number of PI-positive cells increased after ox-LDL treatment (**Supplementary Figure 1F**). Considering that some of the dead cells were discarded from the supernatants, we performed flow cytometry to further confirm the results (**Supplementary Figure 1G**). Our ELISA results showed elevated IL-1β and IL-18 levels in the cell supernatants (**Supplementary Figures 1H, I**). Western blotting showed that the classical pyroptotic pathway was activated, and NLRP3 and ASC expression increased after ox-LDL treatment (**Supplementary Figure 1J**). In addition, the levels of the active forms of these proteins, namely cleaved caspase-1, GSDMD-N, and cleaved IL-1β, significantly increased after ox-LDL exposure (**Supplementary Figure 1J**). GSDMD-N immunofluorescence staining further confirmed this result (**Supplementary Figure 1K**).

To further confirm the effect of cholesterol efflux, we knocked down CD36, a main phagocytic receptor of ox-LDL, to inhibit macrophage phagocytosis, and applied T0901317, an activator of cholesterol excretion receptor, to promote cholesterol excretion. Our results revealed that total cholesterol levels significantly decreased after CD36 knockdown or application of T0901317 (**Supplementary Figure 2A**). In addition, CD36 knockdown or T0901317 application attenuated LDH release into the supernatant, decreased the number of PI-positive cells, and reduced the levels of inflammatory factors such as IL-1β and IL-18 in the cell supernatants (**Supplementary Figures 2B–E**). Western blotting revealed that CD36 knockdown or T0901317 application inhibited the expression of NLRP3 and ASC and prevented the cleavage of caspase-1, GSDMD, and IL-1β (**Supplementary Figure 2F**). These results demonstrated that ox-LDL activated pyroptosis in the macrophages.

### Simvastatin Attenuates Foam Cell Pyroptosis

To confirm the role of simvastatin in foam cell pyroptosis, we added 2 μM simvastatin to a medium containing ox-LDL. Our results suggested that simvastatin inhibited ox-LDL-induced LDH release (**Figure 2B**) and reduced the number of PI-positive cells (**Figures 2C, D**). Microscopic observation also suggested that the number of pyroptotic bubbles decreased in the group treated with simvastatin (**Figure 2E**). ELISA showed that simvastatin reduced IL-1β and IL-18 levels in the cell supernatants (**Figures 2F, G**). Western blotting revealed that NLRP3, ASC, cleaved caspase-1, GSDMD-N, and cleaved IL-1β levels were significantly decreased after simvastatin treatment (**Figure 2H**). GSDMD-N immunofluorescence staining further verified this result (**Figure 2I**). To verify the mechanism underlying the effect of simvastatin on ox-LDL uptake by macrophages, we used Dil-ox-LDL to quantify ox-LDL phagocytosis. However, no significant differences were observed in the quantity of ox-LDL after simvastatin treatment (**Figure 2J**).

### MCC950 Attenuates Foam Cell Pyroptosis

We used 10 μM MCC950, an inhibitor of NLRP3, to verify the role of NLRP3 in foam cell pyroptosis. Our results suggested that MCC950 inhibited LDH release, decreased the number of PI-positive cells, prevented pyroptotic bubble formation, and attenuated IL-1β and IL-18 secretion (**Supplementary Figures 3A–F**). In addition, western blotting revealed that MCC950 inhibited the expression of NLRP3 and ASC and prevented the cleavage of caspase-1, GSDMD, and IL-1β (**Supplementary Figure 3G**). These results were similar to those for simvastatin.

### NLRP3 Overexpression Reverses the Effects of Simvastatin on Foam Cell Pyroptosis

**Figure 3:**
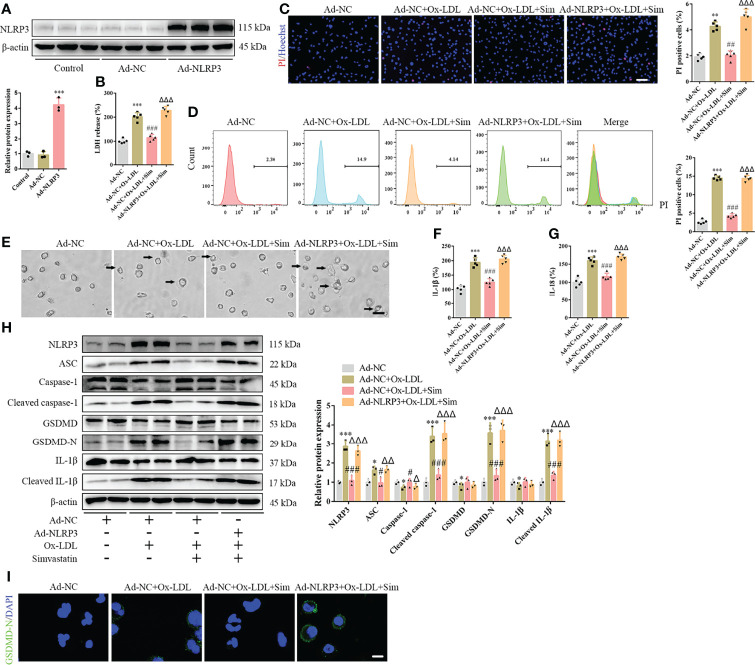
NLRP3 overexpression reverses the effects of simvastatin on foam cell pyroptosis. **(A)** Western blotting showing NLRP3 overexpression. **(B)** Lactate dehydrogenase release in the cell supernatants. **(C)** Hoechst 33342 (blue) and propidium iodide (red) fluorescence staining showing cell membrane integrity. Scale bar, 100 μm. **(D)** Flow cytometry to detect pyroptotic cells. **(E)** Pyroptotic bubbles (arrow) observed through microscopy. Scale bar, 25 μm. **(F, G)** Enzyme-linked immunosorbent assay for the detection of IL-1β and IL-18 levels in the cell supernatants. **(H)** Western blotting showing protein abundance. **(I)** Immunofluorescence staining showing GSDMD-N activation. Scale bar, 12.5 μm. *p < 0.05, **p < 0.01, ***p < 0.001, versus the Ad-NC + ox-LDL group. ^#^ *p* < 0.05, ^##^ *p* < 0.01, ^###^ *p* < 0.001, versus the Ad-NC + ox-LDL group. ^△^ *p* < 0.05, ^△△^ *p* < 0.01, ^△△△^ *p* < 0.001, versus the Ad-NC + ox-LDL + sim group.

To confirm the involvement of the classical pyroptotic pathway in the effects of simvastatin on foam cells, we first established Ad-NLRP3 macrophages (**Figure 3A**). Our results showed that NLRP3 overexpression enhanced LDH release, which was attenuated by simvastatin in foam cells (**Figure 3B**). The effects of simvastatin on the number of PI-positive cells and pyroptotic bubbles and inflammatory cytokine (IL-1β and IL-18) release were reversed after NLRP3 overexpression (**Figures 3C–G**). Correspondingly, western blotting revealed increased levels of NLRP3, ASC, cleaved caspase-1, GSDMD-N, and cleaved IL-1β (**Figures 3H, I**).

### GSDMD Knockdown Attenuates Foam Cell Pyroptosis

To further confirm pyroptotic involvement, we knocked down GSDMD, a pyroptotic performer protein (**Supplementary Figure 4A**). We found that enhanced LDH release induced by ox-LDL was abrogated by GSDMD knockdown (**Supplementary Figure 4B**). Moreover, the number of PI-positive cells, pyroptotic bubbles, and IL-1β and IL-18 levels in the cell supernatants were not elevated after GSDMD knockdown (**Supplementary Figures 4C–G**). However, NLRP3 and ASC expression still increased, and caspase-1 and IL-1β were activated by ox-LDL even after GSDMD knockdown (**Supplementary Figure 4H**).

### Simvastatin Attenuates Foam Cell Pyroptosis by HMGCR

**Figure 4:**
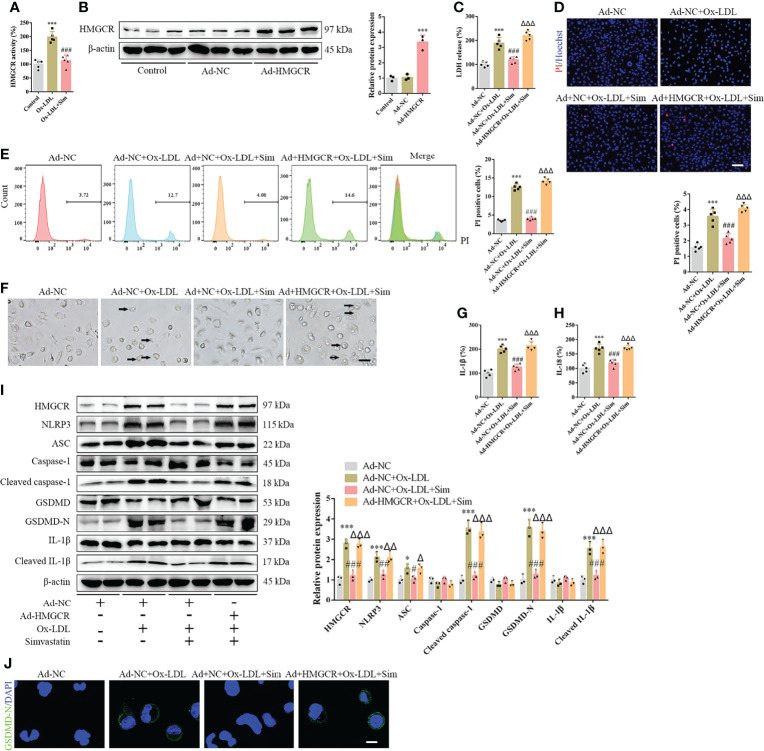
HMGCR overexpression reverses the effects of simvastatin on foam cell pyroptosis. **(A)** HMGCR activity detection. **(B)** Western blotting showing HMGCR overexpression. **(C)** Lactate dehydrogenase release in the cell supernatants. **(D)** Hoechst 33342 (blue) and propidium iodide (red) fluorescence staining showing cell membrane integrity. Scale bar, 100 μm. **(E)** Flow cytometry to detect pyroptotic cells. **(F)** Pyroptotic bubbles (arrow) observed through microscopy. Scale bar, 25 μm. **(G, H)** Enzyme-linked immunosorbent assay for the detection of IL-1β and IL-18 levels in the cell supernatants. **(I)** Western blotting showing protein abundance. **(J)** Immunofluorescence staining showing the GSDMD-N activation. Scale bar, 12.5 μm. *p < 0.05, ***p < 0.001, versus the Ad-NC group. ^#^p < 0.05, ^##^p < 0.01, ^###^p < 0.001, versus the Ad-NC + ox-LDL group. ^△^p < 0.05, ^△△^p < 0.01, ^△△△^p < 0.001, versus the Ad-NC + ox-LDL + sim group.

**Figure 5:**
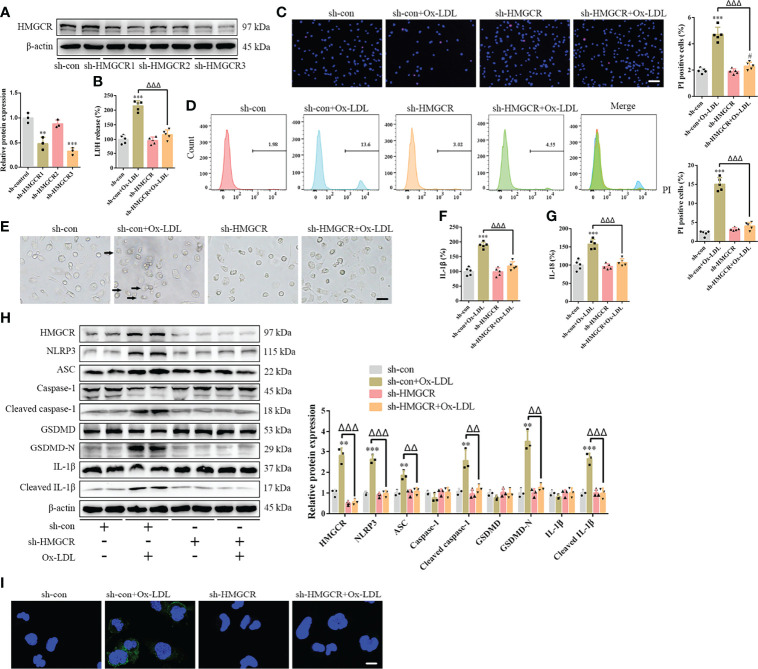
HMGCR knockdown attenuates foam cell pyroptosis. **(A)** Western blotting confirmed HMGCR knockdown. **(B)** Lactate dehydrogenase release in the cell supernatants. **(C)** Hoechst 33342 (blue) and propidium iodide (red) fluorescence staining showing cell membrane integrity. Scale bar, 100 μm. **(D)** Flow cytometry to detect pyroptotic cells. **(E)** Pyroptotic bubbles (arrow) observed through microscopy. Scale bar, 25 μm. **(F, G)** Enzyme-linked immunosorbent assay for the detection of IL-1β and IL-18 levels in the cell supernatants. **(H)** Western blotting showing protein abundance. **(I)** Immunofluorescence staining showing the GSDMD-N activation. Scale bar, 12.5 μm. **p < 0.01, ***p < 0.001, versus the sh-control group. ^#^ *p* < 0.05, versus the sh-HMGCR group. ^△△^ *p* < 0.01, ^△△△^ *p* < 0.001, versus the sh-control + ox-LDL group.

To further study the effects of simvastatin on pyroptosis, three types of statins were used to treat the foam cells. No significant differences were observed among the effects of simvastatin, pravastatin, and lovastatin on foam cell pyroptosis (**Supplementary Figures 5A–G**). Statins inhibit HMGCR, a rate-limiting enzyme in cholesterol synthesis (27). In this study, western blotting showed that HMGCR expression was enhanced after ox-LDL treatment, consistent with previous study results (28), and was attenuated after simvastatin treatment (**Figure 2H**). HMGCR activity detection showed that HMGCR activity was enhanced after ox-LDL treatment and attenuated after simvastatin treatment (**Figure 4A**). Therefore, we overexpressed HMGCR, the known target of statins, to verify the target of simvastatin (**Figure 4B**). Our results showed that HMGCR overexpression reversed the effect of simvastatin (**Figures 4C–J**). Through HMGCR knockdown, we found that ox-LDL-induced pyroptosis was attenuated (**Figures 5A–I**).

### Simvastatin Inhibits the Interaction Between NLRP3 and HMGCR

**Figure 6:**
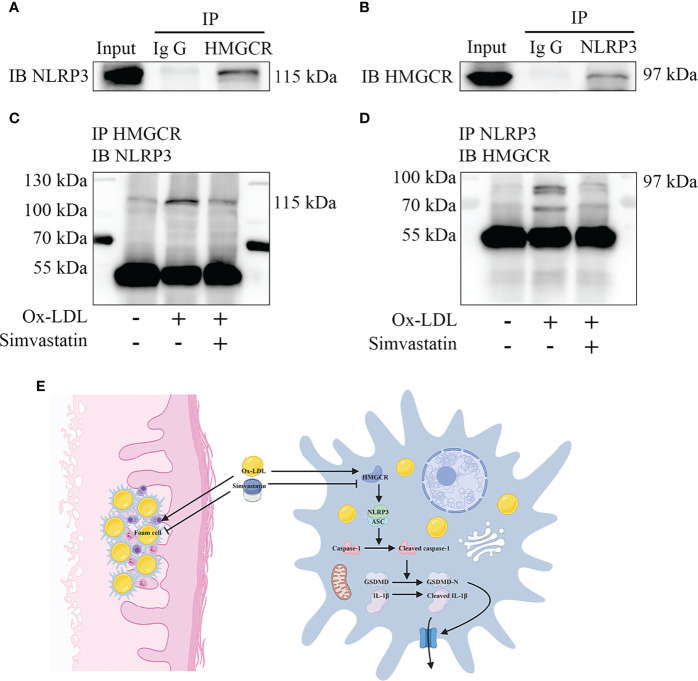
Mechanism underlying the effects of simvastatin. **(A, B)** Immunoprecipitation showed an interaction between NLRP3 and HMGCR. **(C, D)** Simvastatin inhibited the interaction between NLRP3 and HMGCR. **(E)** Schematic model of the effects of simvastatin.

Immunoprecipitation of either NLRP3 or HMGCR led to the recovery of both proteins, supporting the existence of an interaction between NLRP3 and HMGCR (**Figures 6A, B**). Additionally, simvastatin inhibited the interaction between NLRP3 and HMGCR (**Figures 6C, D**). NLRP3 and HMGCR colocalization in DNPX lesions was determined (**Figure 1F**). Our results indicated that simvastatin inhibited inflammation by causing an interaction between NLRP3 and HMGCR. A schematic model of the effects of simvastatin is shown in **Figure 6E**.

## Discussion

To the best of our knowledge, this study is the first to use simvastatin ointment to treat DNPX. The severity of the lesions significantly improved with the 6-month topical simvastatin ointment treatment (**Figure 1D**). Pathologically, there were fewer foam cells and inflammatory cells in the dermis (**Figure 1E**). Although oral lipid-lowering agents generally show good results in patients with DPX, there is no clinically effective treatment for DNPX (12, 14). Furthermore, many statins are only approved for use in patients of 8–10 years of age (29). Therefore, considering normal blood lipid levels and the age limitation of oral statin administration, DNPX treatment for children remains a challenge in clinical practice. Topical treatment is one of the advantages of dermatology, and the concept should be widely applied and advocated. The topical application of simvastatin ointment not only constitutes a potential therapy for patients with DNPX but also avoids the adverse effects of systemic medication.

The causes of lipid deposition in xanthoma are not clear. There have been conjectures, including the presence of qualitatively different lipoproteins at normal plasma lipid concentrations; increased extravasation of lipids (increased vascular permeability, increased local circulation, chronic inflammation); lipid synthesis *in situ* and their deposition; and dysfunction of the reverse cholesterol transport (6). In addition, as xanthoma is accompanied by monoclonal gammopathy in some patients, there is a view that IgG antibodies may participate in the formation of xanthoma by promoting the phagocytosis of LDL by macrophages, which can also explain why some xanthoma patients have normal blood lipid levels (3, 30, 31). Smith described a case of DNPX associated with multiple myeloma, where glucocorticoid therapy relieved IgG proteinemia and the whole-body skin lesions significantly improved (32). Our study was the first to demonstrate ox-LDL deposition (**Figure 2A**) in the cytoplasm of xanthomas. Our results support the hypothesis that xanthomas result from abnormal lipid deposition, which will contribute to the study of xanthoma pathogenesis (2, 6–8, 33).

Using ox-LDL to stimulate macrophages to establish a foam cell model *in vitro*, our study showed that pyroptosis was activated, and various inflammatory cytokines were produced and released, which is consistent with the results of Zhang et al. (24) (**Supplementary Figure 1**). Compared with previous studies, our study further confirmed pyroptosis by observing pyroptotic bubbles and verifying the location of membrane GSDMD-N (**Supplementary Figures 1D, E**). Through CD36 overexpression and T0901317 application, we clarified the role of ox-LDL in pyroptotic activation (**Supplementary Figure 2**). Importantly, our study suggested that simvastatin inhibited ox-LDL-induced inflammation through the classical pyroptotic pathway, consistent with the results obtained for lesion samples (**Figures 1** and **2**). Through knockdown and overexpression of HMGCR, our results indicated that HMGCR played an important role in ox-LDL-induced pyroptosis. Furthermore, our results showed the novel interaction between NLRP3 and HMGCR; this interaction could be attenuated by simvastatin, partially elucidating its mechanism of action (**Figure 6**). Indeed, previous studies have reported that HMGCR is positively related to inflammation (28, 34, 35). In addition, simvastatin reduces cholesterol synthesis by inhibiting HMGCR (27). However, our previous results showed that LDL failed to induce pyroptosis, and cholesterol is only one of the main components of LDL. Therefore, we speculate that the effect of cholesterol is not as remarkable as that of ox-LDL. Ox-LDL has a stronger stimulatory effect; this may be one of the reasons why ox-LDL is used in almost all studies on atherosclerosis (36, 37). Cholesterol is synthesized from acetyl-CoA through a series of approximately 30 reactions, in which many biological changes and numerous molecules and proteins are involved, except HMGCR (38). For example, acetyl-CoA itself is closely related to inflammation (39). Yajing Peng proposed that acetyl-CoA can induce inflammation and cause a progeria-like phenotype (40). Thus, whether the molecules and proteins involved in cholesterol synthesis play a key role in pyroptosis is worth studying.

Inflammation plays an important role in the initiation, development, recovery, and terminal stages of several diseases. Studies have suggested that inflammation also plays an important role in xanthoma (8, 33). Pyroptosis, closely related to inflammation, is involved in not only the production of inflammatory cytokines but also their release (41). Our result that simvastatin improves lesions by inhibiting pyroptosis partially elucidates the therapeutic mechanism, which not only promotes the treatment of DNPX but also contributes to studies on DNPX pathogenesis. It has been reported that the symptoms in some patients with DPX do not improve after blood lipid level reduction by various medicines, which indicates that the pathogenesis is complicated and not solely limited to high blood lipid levels (42, 43). However, in a patient with DNPX with multiple myeloma, the cutaneous symptoms improved significantly, with a reduction in the circulating IgG level, after oral prednisolone treatment (32). Thus, prednisolone, a glucocorticoid, inhibits inflammation, which is consistent with our result that simvastatin improves lesions *via* the inhibition of inflammation.

DNPX not only affects the appearance of patients but is also associated with blood systemic diseases (2, 3, 31). Our results will contribute to studies on these complications. Furthermore, there are several similarities between diseases associated with lipid metabolism, such as DNPX and atherosclerosis, including foam cell aggregation, inflammatory cell infiltration, and tumor formation; additionally, both are chronic diseases. Currently, atherosclerosis is irreversible and creates a huge medical burden (9). However, in our study, simvastatin treatment significantly improved the severity of the lesions of DNPX. Therefore, our results are likely to contribute to studies on the mechanisms underlying simvastatin treatment of atherosclerosis.

There were some limitations to our study. First, DNPX is a complex disease involving not only foam cells but multiple cells, including Touton giant cells and fibroblasts. However, our results were only validated in foam cells. Any one of these cells may also play an important role in the pathogenesis of DNPX, as previous literatures reported that skin fibroblasts can transform to fat cells under certain conditions (44). In addition, our results indicated the existence of an interaction between NLRP3 and HMGCR. However, the specific mechanism underlying this interaction is unknown. Further, we only detected the total GSDMD level rather than the GSDMD-N level in the lesion samples. Although this method has been used in previous studies (19, 45), it is not sufficiently robust for a complete analysis.

In conclusion, we present the topical application of simvastatin as a novel treatment strategy for DNPX. Furthermore, the treatment mechanism is likely related to pyroptotic inhibition through HMGCR in foam cells. However, whether other statins would be similarly effective in humans requires further testing. Our results could contribute to future studies on DNPX and diseases related to foam cells and inflammation.

## Data Availability Statement

The original contributions presented in the study are included in the article/**Supplementary Material**. Further inquiries can be directed to the corresponding authors.

## Ethics Statement

The studies involving human participants were reviewed and approved by Ethics Committee of Xinhua Hospital Affiliated to Shanghai Jiaotong University School of Medicine. Written informed consent to participate in this study was provided by the participants’ legal guardian/next of kin. Written informed consent was obtained from the individual(s), and minor(s)’ legal guardian/next of kin, for the publication of any potentially identifiable images or data included in this article.

## Author Contributions

SZ collected clinical samples, designed, and performed basic experiments, analyzed the results, and wrote the manuscript. XY designed and performed the clinical trials and provided funding support. XW revised the manuscript. YG performed some of the clinical trials. YT performed some of the basic experiments. YL analyzed the results and revised the manuscript. ZY conceived the study and provided funding support. All authors contributed to the article and approved the submitted version.

## Funding

This study was supported by the National Natural Science Foundation of China (grant number 81874252) and the Medical-Engineering Cross Fund of Shanghai Jiao Tong University (grant number YG2021QN57).

## Conflict of Interest

The authors declare that the research was conducted in the absence of any commercial or financial relationships that could be construed as a potential conflict of interest.

## Publisher’s Note

All claims expressed in this article are solely those of the authors and do not necessarily represent those of their affiliated organizations, or those of the publisher, the editors and the reviewers. Any product that may be evaluated in this article, or claim that may be made by its manufacturer, is not guaranteed or endorsed by the publisher.

## Supplementary Material

Supplementary Figure 1 Oxidized low-density lipoprotein (ox-LDL) activates the classical pyroptotic pathway in foam cells. **(A)** Macrophage phagocytosis of Dil-ox-LDL. Scale bar, 100 μm. **(B)** Lactate dehydrogenase release in the cell supernatants in response to various concentrations of ox-LDL. **(C)** Macrophage proliferation under various concentrations of ox-LDL was detected using the CCK8 assay. **(D)** Pyroptotic bubbles (arrow) observed under the microscope. Scale bar, 25 μm. **(E)** Confocal microscopy showing GSDMD-N location. Scale bar, 12.5 μm. **(F)** Hoechst 33342 (blue) and propidium iodide (red) fluorescence staining show cell membrane integrity. Scale bar, 100 μm. **(G)** Flow cytometry to detect pyroptotic cells. **(H, I)** Enzyme-linked immunosorbent assay for the detection of IL-1β and IL-18 levels in the cell supernatants. **(J)** Western blotting showing protein abundance. **(K)** Immunofluorescence staining showing GSDMD-N activation. Scale bar, 12.5 μm. *p < 0.05, **p < 0.01, ***p < 0.001, versus the control.

Supplementary Figure 2 CD36 knockdown and application of T0901317 attenuated oxidized low-density lipoprotein (ox-LDL)-induced pyroptosis. **(A)** Total cholesterol levels in the macrophages. **(B)** Lactate dehydrogenase release in the cell supernatants. **(C)** Flow cytometry to detect pyroptotic cells. **(D, E)** Enzyme-linked immunosorbent assay for the detection of IL-1β and IL-18 levels in the cell supernatants. **(F)** Western blotting showing protein abundance. *p < 0.05, ***p < 0.001, versus the control. ^##^p < 0.01, ^###^p < 0.001, versus the sh-control + ox-LDL group.

Supplementary Figure 3 MCC950 attenuates foam cell pyroptosis. **(A)** Lactate dehydrogenase release in the cell supernatants. **(B)** Hoechst 33342 (blue) and propidium iodide (red) staining show cell membrane integrity. Scale bar, 100 μm. **(C)** Flow cytometry for the detection of pyroptotic cells. **(D)** Pyroptotic bubbles (arrow) observed under the microscope. Scale bar, 25 μm. **(E, F)** Enzyme-linked immunosorbent assay for the detection of IL-1β and IL-18 levels in the cell supernatants. **(G)**. Western blotting showing protein levels. *p < 0.05, **p < 0.01, ***p < 0.001, versus the control. ^#^p < 0.05, ^##^p < 0.01, ^###^p < 0.001, versus the ox-LDL group.

Supplementary Figure 4 GSDMD knockdown attenuates foam cell pyroptosis. **(A)** Western blotting confirmed GSDMD knockdown. **(B)** Lactate dehydrogenase release in the cell supernatants. **(C)** Hoechst 33342 (blue) and propidium iodide (red) fluorescence staining showing cell membrane integrity. Scale bar, 100 μm. **(D)** Flow cytometry to detect pyroptotic cells. **(E)** Pyroptotic bubbles (arrow) observed under the microscope. Scale bar, 25 μm. **(F, G)** Enzyme-linked immunosorbent assay for the detection of IL-1β and IL-18 levels in the cell supernatants. **(H)** Western blotting showing protein abundance. *p < 0.05, **p < 0.01, ***p < 0.001, versus the sh-control group. ^#^p < 0.05, ^##^p < 0.01, versus the sh-GSDMD group. ^△△^p < 0.01, ^△△△^p < 0.001, versus the sh-control + ox-LDL group.

Supplementary Figure 5 Statins attenuate foam cell pyroptosis. **(A)** Lactate dehydrogenase release in the cell supernatants. **(B)** Hoechst 33342 (blue) and propidium iodide (red) staining show cell membrane integrity. Scale bar, 100 μm. **(C)** Flow cytometry to detect pyroptotic cells. **(D)** Pyroptotic bubbles (arrow) observed under the microscope. Scale bar, 25 μm. **(E, F)** Enzyme-linked immunosorbent assay for the detection of IL-1β and IL-18 levels in the cell supernatants. **(G)** Western blotting showing protein abundance. *p < 0.05, **p < 0.01, ***p < 0.001, versus the control.
