# Supplementary figures and images for: Topical Simvastatin Improves Lesions of Diffuse Normolipemic Plane Xanthoma by Inhibiting Foam Cell Pyroptosis
Source: Front Immunol. 2022 May 10;13:865704. doi: 10.3389/fimmu.2022.865704 (PMC9128406; doi:10.3389/fimmu.2022.865704)

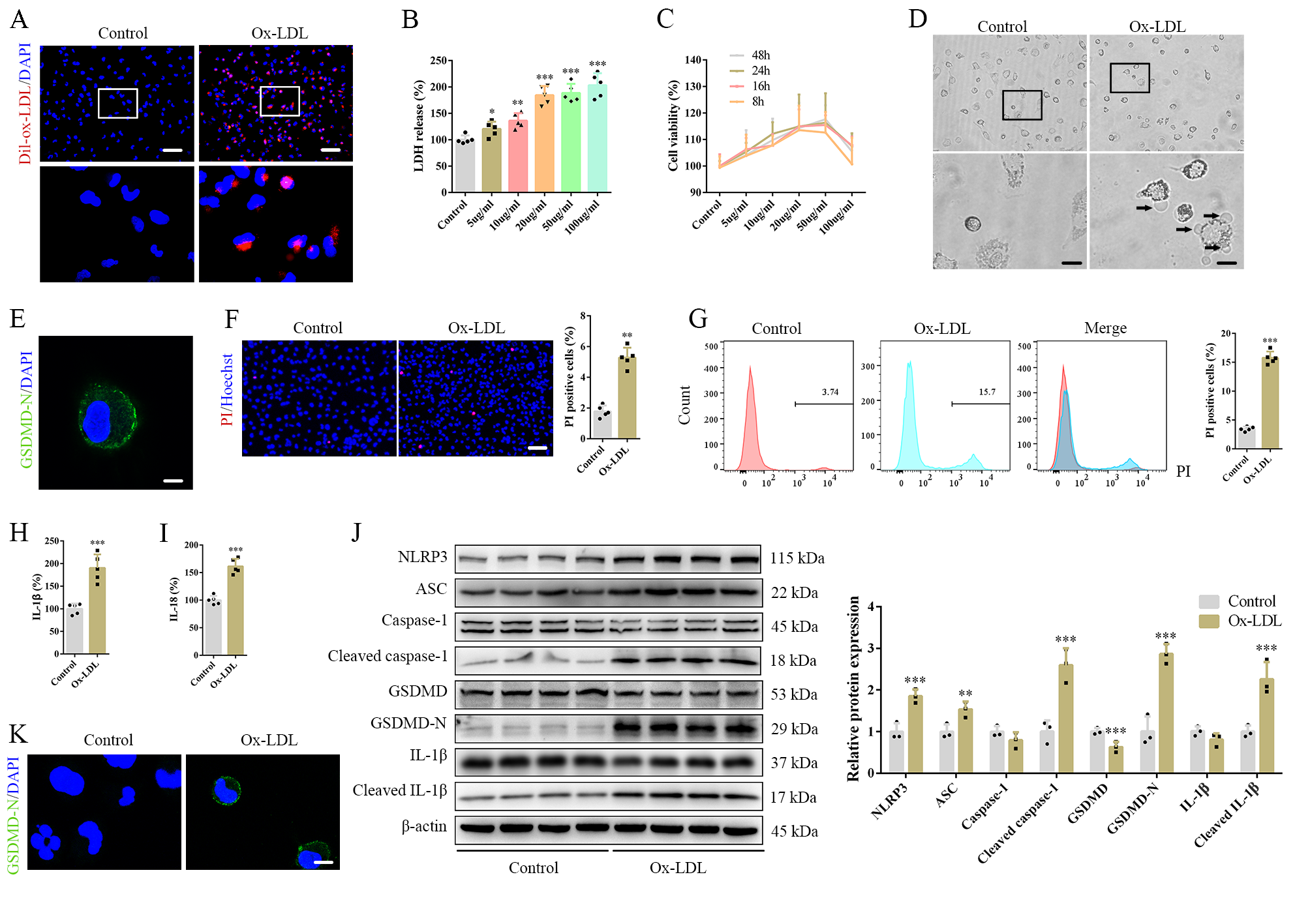

Supplement: Supplementary Figure 1 — Oxidized low-density lipoprotein (ox-LDL) activates the classical pyroptotic pathway in foam cells. (A) Macrophage phagocytosis of Dil-ox-LDL. Scale bar, 100 μm. (B) Lactate dehydrogenase release in the cell supernatants in response to various concentrations of ox-LDL. (C) Macrophage proliferation under various concentrations of ox-LDL was detected using the CCK8 assay. (D) Pyroptotic bubbles (arrow) observed under the microscope. Scale bar, 25 μm. (E) Confocal microscopy showing GSDMD-N location. Scale bar, 12.5 μm. (F) Hoechst 33342 (blue) and propidium iodide (red) fluorescence staining show cell membrane integrity. Scale bar, 100 μm. (G) Flow cytometry to detect pyroptotic cells. (H, I) Enzyme-linked immunosorbent assay for the detection of IL-1β and IL-18 levels in the cell supernatants. (J) Western blotting showing protein abundance. (K) Immunofluorescence staining showing GSDMD-N activation. Scale bar, 12.5 μm. *p < 0.05, **p < 0.01, ***p < 0.001, versus the control. [file Image_1.tif]

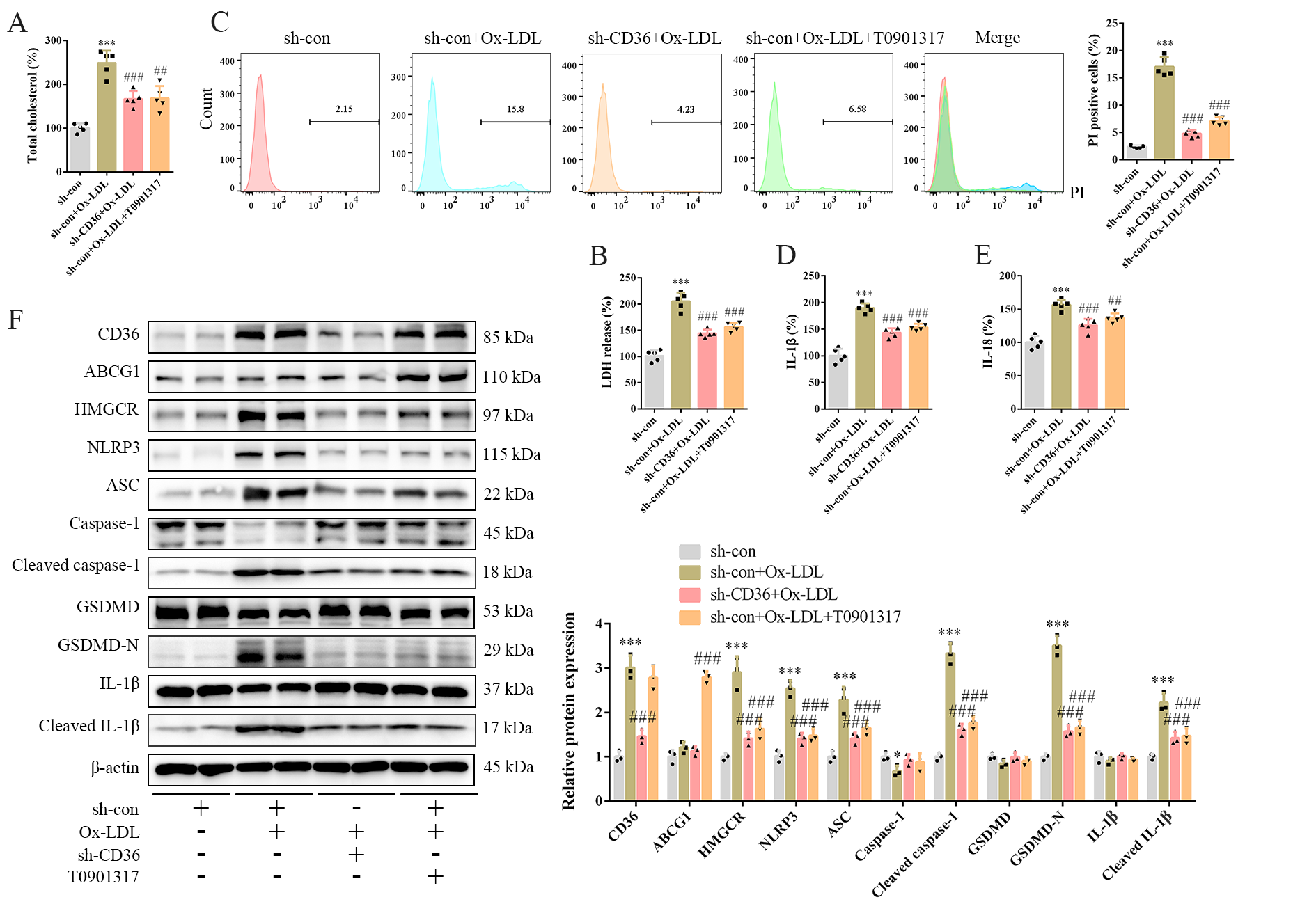

Supplement: Supplementary Figure 2 — CD36 knockdown and application of T0901317 attenuated oxidized low-density lipoprotein (ox-LDL)-induced pyroptosis. (A) Total cholesterol levels in the macrophages. (B) Lactate dehydrogenase release in the cell supernatants. (C) Flow cytometry to detect pyroptotic cells. (D, E) Enzyme-linked immunosorbent assay for the detection of IL-1β and IL-18 levels in the cell supernatants. (F) Western blotting showing protein abundance. *p < 0.05, ***p < 0.001, versus the control. ##p < 0.01, ###p < 0.001, versus the sh-control + ox-LDL group. [file Image_2.tif]

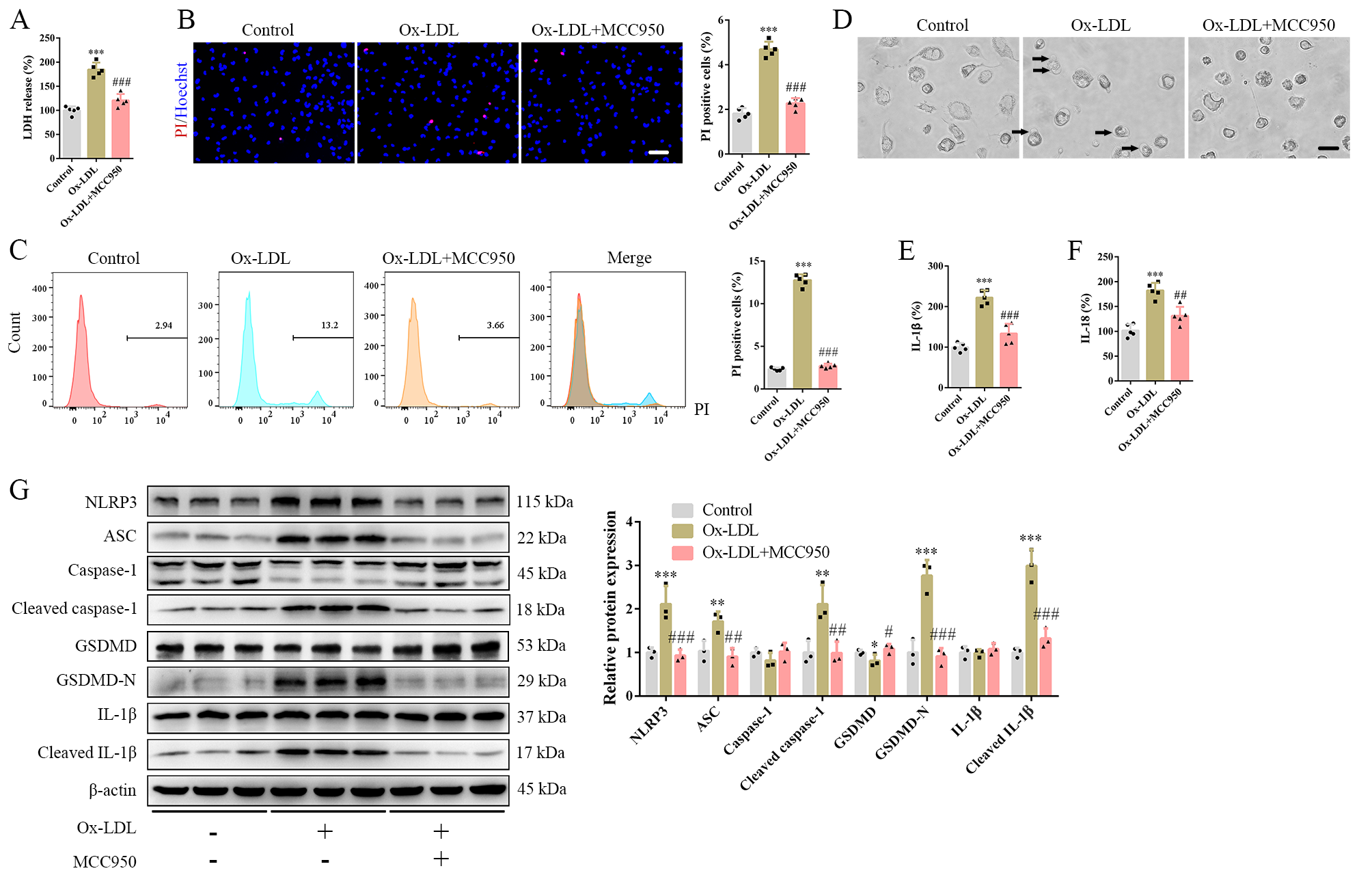

Supplement: Supplementary Figure 3 — MCC950 attenuates foam cell pyroptosis. (A) Lactate dehydrogenase release in the cell supernatants. (B) Hoechst 33342 (blue) and propidium iodide (red) staining show cell membrane integrity. Scale bar, 100 μm. (C) Flow cytometry for the detection of pyroptotic cells. (D) Pyroptotic bubbles (arrow) observed under the microscope. Scale bar, 25 μm. (E, F) Enzyme-linked immunosorbent assay for the detection of IL-1β and IL-18 levels in the cell supernatants. (G). Western blotting showing protein levels. *p < 0.05, **p < 0.01, ***p < 0.001, versus the control. #p < 0.05, ##p < 0.01, ###p < 0.001, versus the ox-LDL group. [file Image_3.tif]

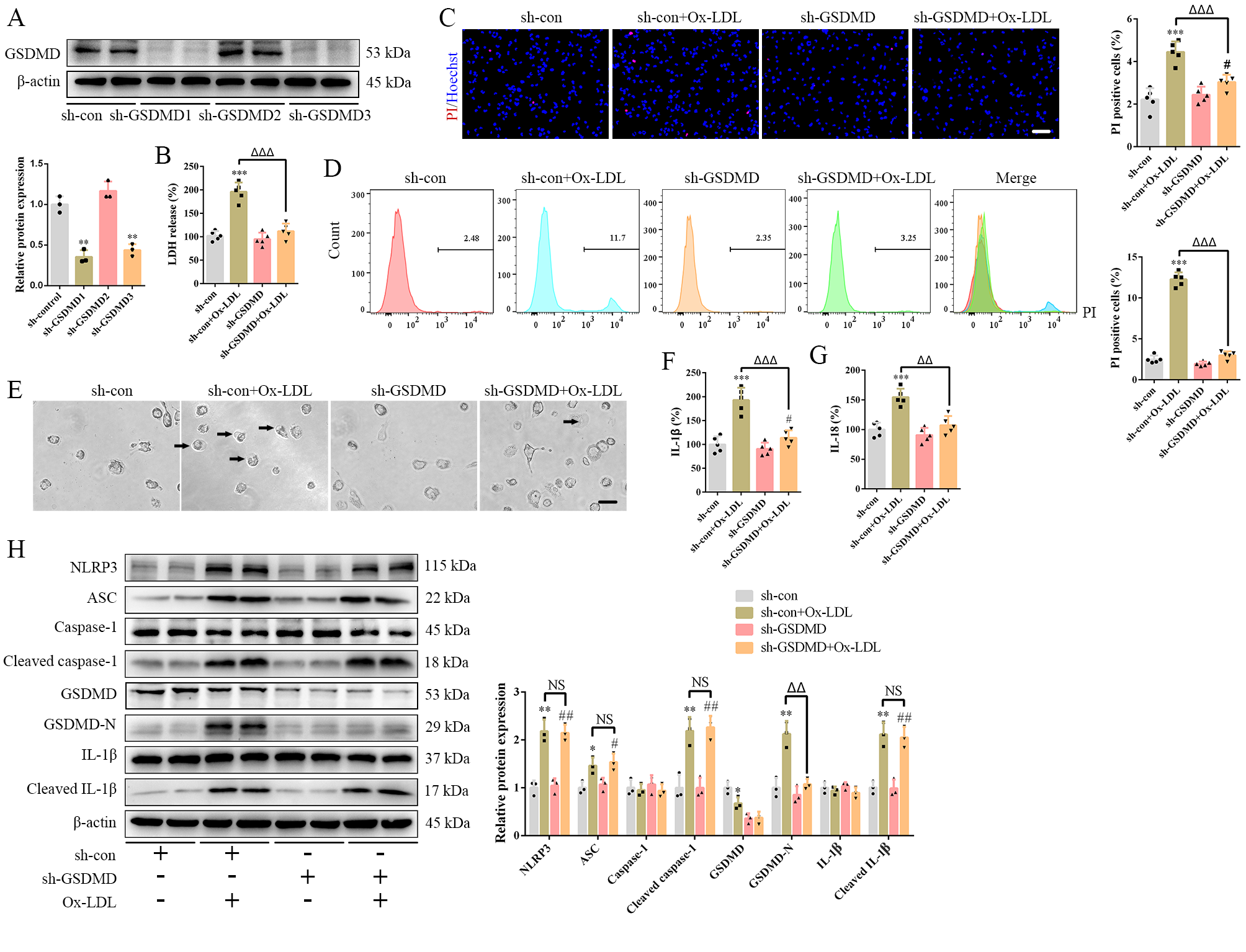

Supplement: Supplementary Figure 4 — GSDMD knockdown attenuates foam cell pyroptosis. (A) Western blotting confirmed GSDMD knockdown. (B) Lactate dehydrogenase release in the cell supernatants. (C) Hoechst 33342 (blue) and propidium iodide (red) fluorescence staining showing cell membrane integrity. Scale bar, 100 μm. (D) Flow cytometry to detect pyroptotic cells. (E) Pyroptotic bubbles (arrow) observed under the microscope. Scale bar, 25 μm. (F, G) Enzyme-linked immunosorbent assay for the detection of IL-1β and IL-18 levels in the cell supernatants. (H) Western blotting showing protein abundance. *p < 0.05, **p < 0.01, ***p < 0.001, versus the sh-control group. #p < 0.05, ##p < 0.01, versus the sh-GSDMD group. △△p < 0.01, △△△p < 0.001, versus the sh-control + ox-LDL group. [file Image_4.tif]

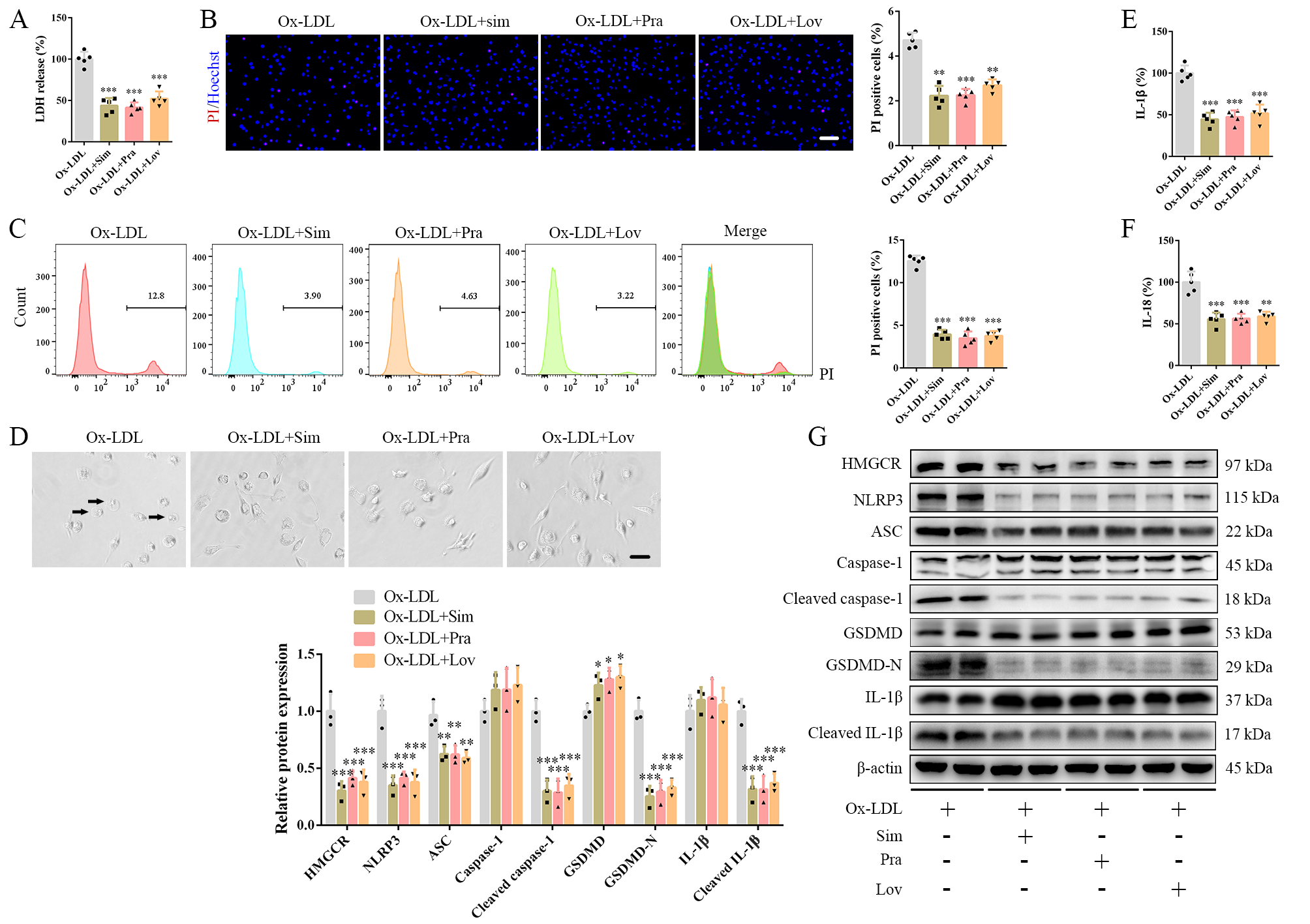

Supplement: Supplementary Figure 5 — Statins attenuate foam cell pyroptosis. (A) Lactate dehydrogenase release in the cell supernatants. (B) Hoechst 33342 (blue) and propidium iodide (red) staining show cell membrane integrity. Scale bar, 100 μm. (C) Flow cytometry to detect pyroptotic cells. (D) Pyroptotic bubbles (arrow) observed under the microscope. Scale bar, 25 μm. (E, F) Enzyme-linked immunosorbent assay for the detection of IL-1β and IL-18 levels in the cell supernatants. (G) Western blotting showing protein abundance. *p < 0.05, **p < 0.01, ***p < 0.001, versus the control. [file Image_5.tif]
