# Supplementary material for: Topical Simvastatin Improves Lesions of Diffuse Normolipemic Plane Xanthoma by Inhibiting Foam Cell Pyroptosis
Source: Front Immunol. 2022 May 10;13:865704. doi: 10.3389/fimmu.2022.865704 (PMC9128406; doi:10.3389/fimmu.2022.865704)
Supplement: Supplementary file 6 [file Table_1.docx]

Baseline information of the participants

| ID | Gender | Age (Year) | Disease type | Total cholesterol  (mmol/L) | Total triglyceride (mmol/L) | HDL  (mmol/L) | LDL  (mmol/L) |
| --- | --- | --- | --- | --- | --- | --- | --- |
| 1 | M | 1 | DNPX | 3.98 (3.1-5.2) | 0.74 (0.68-1.88) | 1.27(0.92-2.59) | 2.68(0-3.12) |
| 2 | M | 2 | DNPX | 2.17 (3.1-5.2) | 0.60 (0.68-1.88) | 1.22(0.92-2.59） | 2.21(0-3.12) |
| 3 | F | 3 | DNPX | 3.47 (3.1-5.2) | 1.45 (0.68-1.88) | 1.09(0.92-2.59) | 1.86(0-3.12) |
